# Seasonal ambient temperature and particulate matter as discriminators of hyperemesis gravidarum severity: a retrospective comparative observational study

**DOI:** 10.3389/fpubh.2026.1957434

**Published:** 2026-09-29

**Authors:** Şahide Akbulut, Erhan Okuyan, Mülaim Sizer, İlyas Turan, Emre Günakan

**Affiliations:** 1 Department of Nursing, Faculty of Health Sciences, Batman University, Batman, Türkiye; 2 Department of Obstetrics and Gynecology, Private Zilan Hospital, Batman, Türkiye; 3 Department of Obstetrics and Gynecology, Batman Training and Research Hospital, Batman, Türkiye; 4 School of Medicine, Atılım University, Ankara, Türkiye

**Keywords:** ambient temperature, environmental exposure, hyperemesis gravidarum, ketonuria, pregnancy

## Abstract

**Introduction:**

Ambient air pollutants and temperature may influence the clinical severity of hyperemesis gravidarum (HG), but their relative contributions remain unclear. This study aimed to determine whether particulate matter (PM₁₀), sulfur dioxide (SO₂), and ambient temperature were independently associated with severe ketonuria in pregnant women with HG.

**Methods:**

This retrospective observational study included 211 pregnant women diagnosed with HG. Clinical severity was defined by ketonuria category (severe ≥2 + vs. non-severe <2+). Covariates (maternal age, gestational week, BMI, Healthy Eating Index [HEI], gravida, PM₁₀, SO₂, and ambient temperature) were selected *a priori* based on literature and univariate screening. Multivariable binary logistic regression was used; model discrimination was assessed via ROC analysis.

**Results:**

The autumn/winter group showed significantly higher maternal age, gravida, and PM₁₀ exposure, and lower ambient temperature than the spring/summer group (all *p* < 0.001). In multivariable analysis, only lower ambient temperature (OR = 0.890, 95% CI: 0.845–0.938, *p* < 0.001) and higher maternal age (OR = 1.143, 95% CI: 1.057–1.237, *p* < 0.001) were independently associated with severe ketonuria; PM₁₀, SO₂, HEI, gestational week, BMI, and gravida were not significant (all *p* > 0.05). The model correctly classified 81.5% of cases (Nagelkerke *R*^2^ = 0.426). ROC analysis confirmed ambient temperature (AUC = 0.799) and PM₁₀ (AUC = 0.754) as significant discriminators for severe versus non-severe ketonuria, while SO₂ was not (AUC = 0.532).

**Conclusion:**

Ambient temperature showed the strongest discriminative performance for HG severity among the environmental parameters examined, while the association of PM₁₀ was attenuated after adjustment for temperature. Maternal age was also independently associated with HG severity. These findings should be interpreted as associations rather than causal effects. Further prospective, multi-site studies incorporating individual-level exposure assessments are warranted to confirm these associations and elucidate the underlying mechanisms.

## 1 Introduction

“Hyperemesis gravidarum (HG)” is a severe form of nausea and vomiting of pregnancy that affects 0.3 to 3% of pregnancies (1–3, 25). Although the aetiology remains unclear, hormonal changes, particularly elevated human chorionic gonadotropin (hCG) levels, are strongly implicated (4). Early recognition and treatment are essential to prevent maternal and fetal complications (5–7). Recent studies have investigated the relationship between air pollution and adverse pregnancy outcomes, such as preterm birth, low birth weight, and gestational diabetes (8–10, 37). However, evidence directly linking air pollutants to HG is scarce and inconsistent. A large population-based cohort study (*n* ≈ 3.7 million) identified sulfur dioxide (SO₂) as the primary pollutant contributing to HG risk (11), while a Mendelian randomization study found no causal effect for particulate matter (PM₁₀) on adverse outcomes (12). Notably, these studies focused on HG risk, not severity, and did not examine meteorological factors.

Meteorological factors, especially ambient temperature, have been associated with various pregnancy complications. For instance, extreme heat and cold have been linked to preterm birth and gestational hypertension (13, 14). Seasonal variations in HG symptoms have been reported, with some studies suggesting higher hospitalization rates during colder months (15, 16). However, few studies have simultaneously examined the independent contributions of air pollution and temperature to HG severity, particularly in industrial settings with high pollution levels.

Given the contradictory findings and the lack of integrated environmental assessments, this study aimed to evaluate whether ambient temperature, PM₁₀, and SO₂ were independently associated with the clinical severity of HG, measured by ketonuria severity, after adjusting for maternal characteristics and nutritional status. We specifically tested the hypothesis that cold temperature, rather than individual pollutants, would be the dominant environmental factor associated with increased HG severity in a polluted urban environment.

## 2 Methods

### 2.1 Study design and setting

This single-center retrospective comparative study was conducted at Batman Maternity and Child Health Hospital, Batman, Türkiye, covering the period from September 2024 to August 2025. The study was reported in accordance with the STROBE guidelines. Patient records were reviewed for women admitted with a diagnosis of HG. The study aimed to evaluate the association between environmental factors and HG severity across two contrasting seasonal periods.

### 2.2 Ethical approval and consent

The study was conducted in accordance with the Helsinki Declaration. Ethical approval was obtained from the Batman University Scientific Research and Publication Ethics Committee (Decision No: 2026/01–43, January 8, 2026). The requirement for written informed consent was waived due to the retrospective nature of the study. Patient confidentiality was maintained throughout.

### 2.3 Environmental data and exposure assessment

Daily air pollution and meteorological data were obtained from the Batman Central Monitoring Station (Station ID: TR-72-001; coordinates: 37.8812°N, 41.1351°E), operated by the Ministry of Environment, Urbanization and Climate Change of Türkiye (29). The station is located approximately 3 km from the hospital. PM₁₀ was measured using beta attenuation (Thermo Scientific TEOM 1405-F), SO₂ by ultraviolet fluorescence (Thermo Scientific 43i-TLE), and ambient temperature by a standard meteorological sensor. All measurements represented 24-h mean concentrations. Quality control included regular calibration and data validation (33). Missing data (<2%) were imputed by linear interpolation. These data are publicly available and updated weekly at.^1^ Daily environmental values were matched to each patient’s admission date. Patients were grouped into two seasons: Autumn/Winter (September 2024–February 2025) and Spring/Summer (March 2025–August 2025).

### 2.4 Study population and criteria

We included pregnant women between 6 and 13 gestational weeks, diagnosed with HG, requiring hospitalization due to nausea, vomiting, and ketonuria. Exclusion criteria were: obesity (BMI > 30 kg/m^2^), absence of ketonuria, abnormal clinical findings (e.g., chorioamnionitis, fetal distress), anemia, leukocytosis (>10,000/mm^3^), and thyroid dysfunction. The final cohort comprised 211 women.

### 2.5 Clinical and nutritional assessment

The primary outcome was HG severity, defined by ketonuria levels on admission: severe (≥2+) versus non-severe (<2+). Treatment duration was recorded as ≤3 days, 3–6 days, or >6 days.

Nutritional status was assessed using the Healthy Eating Index-2015 (HEI-2015) adapted for Turkish pregnant women (15, 17). The HEI includes 13 components (total fruits, whole fruits, vegetables, greens and beans, whole grains, dairy, total protein foods, seafood and plant proteins, fatty acid ratio, refined grains, sodium, added sugars, and saturated fats), each scored 0–10 or 0–20, summed to a total score of 0–100. A cut-off of 90 was used to categorize dietary quality, based on the distribution in the study sample and prior literature. Twenty-four-hour dietary recalls were extracted from admission records.

### 2.6 Statistical analysis

All analyses were performed using SPSS version 26.0. Normality of continuous variables was assessed using the Shapiro–Wilk test; none were normally distributed, so non-parametric tests were used. Comparisons between seasonal groups were made with the Mann–Whitney *U* test for continuous variables and the Chi-square test for categorical variables. For trends across gestational-week subgroups, the linear-by-linear association test was used.

Covariates for the multivariable model were selected based on a combination of clinical relevance and univariate screening (variables with *p* < 0.20 in bivariate analyses). The final model included maternal age, gestational week, BMI, HEI score, gravida, PM₁₀, SO₂, and ambient temperature. Multicollinearity was assessed using variance inflation factor (VIF) and tolerance; the Box-Tidwell test was used to verify linearity in the logit. Multivariable binary logistic regression (enter method) was performed to identify independent predictors of severe ketonuria. Model fit was evaluated with the Hosmer-Lemeshow test, and discrimination with ROC analysis. We additionally report sensitivity, specificity, positive predictive value, negative predictive value, and AUC at the optimal cut-off (Youden’s J). Statistical significance was set at *p* < 0.05.

## 3 Results

### 3.1 Baseline characteristics

Table 1 summarizes the demographic, clinical, and environmental characteristics of the study cohort. The mean age was 27.00 ± 4.91 years, and the mean body mass index (BMI) was 20.94 ± 1.30 kg/m^2^, indicating a predominantly young, normal-weight population. The mean Healthy Eating Index (HEI) score was 87.18 ± 4.05, suggesting generally favorable dietary quality. Regarding environmental exposures, the mean ambient temperature was 19.78 ± 9.45 °C, mean PM₁₀ concentration was 82.79 ± 28.73 μg/m^3^, and mean SO₂ concentration was 4.71 ± 0.88 μg/m^3^.

**Table 1:** Baseline demographic, clinical, and environmental characteristics of the study cohort (*N* = 211).

Characteristic	Overall (*N* = 211)
Continuous variables	Mean ± SD (Min–Max)
Age (years)	27.00 ± 4.91 (17.00–42.00)
BMI (kg/m^2^)	20.94 ± 1.30 (18.00–29.00)
HEI score	87.18 ± 4.05 (80.00–100.00)
Ambient temperature (°C)	19.78 ± 9.45 (3.60–45.50)
PM₁₀ (μg/m^3^)	82.79 ± 28.73 (30.86–172.18)
SO₂ (μg/m^3^)	4.71 ± 0.88 (2.55–9.02)

Categorical variables	*n* (%)
Ketonuria category	
Non-severe (<2+)	119 (56.4%)
Severe (≥2+)	92 (43.6%)
Gestational age (Weeks)	
6–7	48 (22.7%)
8–9	76 (36.0%)
10–11	62 (29.4%)
12–13	25 (11.8%)
Treatment duration	
≤3 days	75 (35.5%)
3–6 days	76 (36.0%)
>6 days	60 (28.4%)
Season (Period)	
Autumn/Winter	90 (42.7%)
Spring/Summer	121 (57.3%)

Continuous variables are presented as Mean ± SD (Min–Max); categorical variables as *n* (%). SD, Standard Deviation; Min, Minimum; Max, Maximum; BMI, Body Mass Index; HEI, Healthy Eating Index; PM₁₀, Particulate Matter ≤10 μm; SO₂, Sulfur Dioxide.

Severe ketonuria (≥2+) was present in 43.6% (n = 92) of participants. The largest proportion presented at 8–9 weeks of gestation (36.0%), and most required 3–6 days of treatment (36.0%). More than half of the participants presented during the spring/summer period (57.3%).

### 3.2 Seasonal group comparisons

Table 2 compares clinical and demographic variables between participants presenting during the autumn/winter period (Group 1, *n* = 90) and the spring/summer period (Group 2, *n* = 121). Group 1 participants were significantly older (28.73 ± 4.84 vs. 25.70 ± 4.56 years; *p* < 0.001), had later gestational age (9.58 ± 2.13 vs. 8.74 ± 1.52 weeks; *p* = 0.002), and had higher gravida (3.96 ± 0.98 vs. 2.68 ± 0.99; *p* < 0.001). Ambient temperature was markedly lower in Group 1 (11.45 ± 3.24 °C vs. 25.98 ± 7.59 °C; *p* < 0.001), and PM₁₀ was significantly higher (104.39 ± 22.62 vs. 66.72 ± 21.33 μg/m^3^; *p* < 0.001). No significant differences were observed for BMI, HEI score, or SO₂.

**Table 2:** Comparison of Clinical and Demographic Variables by Seasonal Group.

Variable	Group 1 (Autumn/Winter) Mean ± SD	Group 2 (Spring/Summer) Mean ± SD	t (df)	*p*-value	Cohen’s *d*
Age (years)	28.73 ± 4.84	25.70 ± 4.56	4.65 (209)	<0.001*	0.65
Gestational age (weeks)	9.58 ± 2.13	8.74 ± 1.52	3.19 (152.95)ᵃ	0.002*	0.46
Gravida (*n*)	3.96 ± 0.98	2.68 ± 0.99	9.33 (209)	<0.001*	1.30
BMI (kg/m^2^)	21.01 ± 1.16	20.89 ± 1.40	0.66 (209)	0.513	0.09
HEI score	87.40 ± 3.97	87.01 ± 4.11	0.69 (209)	0.488	0.10
Ambient temperature (°C)	11.45 ± 3.24	25.98 ± 7.59	−18.87 (172.10)ᵃ	<0.001*	−2.37
PM₁₀ (μg/m^3^)	104.39 ± 22.62	66.72 ± 21.33	12.37 (209)	<0.001*	1.72
SO₂ (μg/m^3^)	4.76 ± 1.00	4.68 ± 0.79	0.64 (164.81)ᵃ	0.524	0.09

SD, Standard deviation; df, Degrees of freedom; BMI, Body Mass Index; HEI, Healthy Eating Index; PM₁₀, Particulate Matter ≤10 μm; SO₂, Sulfur dioxide. ᵃWelch’s *t*-test was applied due to violation of the homogeneity of variances assumption (Levene’s test *p* < 0.05). *p < 0.01.

### 3.3 Distribution across gestational-week subgroups

Table 3 presents the distribution of season, HEI2 measurement, treatment duration, and ketonuria category across gestational-week subgroups. Season of admission (χ^2^ = 19.944, *p* < 0.001), HEI2 (χ^2^ = 13.554, *p* = 0.004), treatment duration (χ^2^ = 24.777, *p* < 0.001), and ketonuria severity (χ^2^ = 13.247, *p* = 0.004) were all significantly associated with gestational week. The linear-by-linear association test confirmed significant linear trends for all four variables (*p* < 0.05 for all).

**Table 3:** Distribution of admission period, HEI2 second measurement, treatment duration, and ketonuria category by Gestational-Week subgroup (*N* = 211).

Variable	Category	6–7 weeks (*n* = 48) *n* (%)	8–9 weeks (*n* = 76) *n* (%)	10–11 weeks (*n* = 62) *n* (%)	12–13 weeks (*n* = 25) *n* (%)	χ^2^ (df)	*p*-value
Season	Autumn/Winter	17 (35.4)	28 (36.8)	24 (38.7)	21 (84.0)	19.944 (3)	<0.001**
	Spring/Summer	31 (64.6)	48 (63.2)	38 (61.3)	4 (16.0)		
HEI	<90	42 (87.5)	57 (75.0)	37 (59.7)	14 (56.0)	13.554 (3)	0.004*
	≥90	6 (12.5)	19 (25.0)	25 (40.3)	11 (44.0)		
Treatment duration	≤3 days	25 (52.1)	33 (43.4)	14 (22.6)	3 (12.0)	24.777 (6)	<0.001**
	3–6 days	13 (27.1)	19 (25.0)	33 (53.2)	11 (44.0)		
	>6 days	10 (20.8)	24 (31.6)	15 (24.2)	11 (44.0)		
Ketonuria category	Non-severe (<2+)	26 (54.2)	48 (63.2)	39 (62.9)	6 (24.0)	13.247 (3)	0.004*
	Severe (≥2+)	22 (45.8)	28 (36.8)	23 (37.1)	19 (76.0)		

Values are presented as *n* (column %). Pearson Chi-Square (χ^2^) test was performed. Linear-by-linear association test: Season *p* < 0.001; HEI2 *p* = 0.002; Ketonuria *p* = 0.002. *p < 0.01; **p < 0.001.

### 3.4 Correlation analyses

Table 4 (patient-level, *N* = 211) shows that ketonuria severity was positively correlated with maternal age (*r_s_* = 0.203, *p* = 0.003), gestational week (*r_s_* = 0.138, *p* = 0.045), gravida (r_s_ = 0.450, *p* < 0.001), and treatment duration (*r_s_* = 0.463, *p* < 0.001).

**Table 4:** Spearman’s rho correlation analysis among clinical and nutritional variables (patient-level, *N* = 211).

Variable	1	2	3	4	5	6	7
1. Maternal age	—						
2. Gestational week	−0.056	—					
3. BMI	0.032	0.031	—				
4. HEI	−0.078	0.232**	−0.087	—			
5. Ketonuria severity	0.203**	0.138*	0.031	0.089	—		
6. Gravida	0.114	0.282**	0.073	0.101	0.450**	—	
7. Treatment duration	0.137*	0.232**	−0.044	−0.008	0.463**	0.510**	—

Values represent Spearman’s rank correlation coefficient (*r_s_*). *N* = 211 for all pairs. HEI, Healthy Eating Index. *p < 0.05; **p < 0.01.

Table 5 (environmental time-series, *N* = 365) shows that ambient temperature was negatively correlated with PM₁₀ (*r_s_* = −0.391, *p* < 0.001), while its correlation with SO₂ was not statistically significant (*r_s_* = 0.050, *p* = 0.344). PM₁₀ was positively correlated with SO₂ (*r_s_* = 0.153, *p* = 0.003).

**Table 5:** Spearman’s rho correlation analysis among environmental variables (time-series, *N* = 365).

Variable	1	2	3
1. Ambient temperature	—		
2. PM₁₀	−0.391**	—	
3. SO₂	0.050	0.153**	—

Values represent Spearman’s rank correlation coefficient (*r_s_*). *N* = 365 for all pairs. PM₁₀, Particulate Matter ≤10 μm; SO₂, Sulfur dioxide. *p < 0.05; **p < 0.01.

### 3.5 Multivariable logistic regression

Table 6 presents the multivariable binary logistic regression model for severe ketonuria (≥2+). After adjustment for all covariates, only maternal age (OR = 1.143, 95% CI: 1.057–1.237, *p* < 0.001) and ambient temperature (OR = 0.890, 95% CI: 0.845–0.938, *p* < 0.001) were independently associated with severe ketonuria. For each 1 °C increase in ambient temperature, the odds of severe ketonuria decreased by approximately 11%. Gestational week, BMI, HEI, gravida, PM₁₀, and SO₂ were not significant (*p* > 0.05 for all).

**Table 6:** Multivariable binary logistic regression model for independent predictors of severe ketonuria (≥2+).

Variable	B	S.E.	Wald	*p*-value	OR (95% CI)
Maternal age (years)	0.134	0.040	11.160	<0.001**	1.143 (1.057–1.237)
Gestational week	−0.051	0.097	0.281	0.596	0.950 (0.786–1.148)
BMI (kg/m^2^)	−0.124	0.137	0.816	0.366	0.883 (0.675–1.156)
HEI score	0.047	0.043	1.208	0.272	1.048 (0.964–1.139)
Gravida (n)	0.235	0.177	1.752	0.186	1.264 (0.893–1.789)
PM₁₀ (μg/m^3^)	0.008	0.008	0.939	0.333	1.008 (0.992–1.024)
Ambient temperature (°C)	−0.116	0.026	19.261	<0.001**	0.890 (0.845–0.938)
SO₂ (μg/m^3^)	0.166	0.201	0.682	0.409	1.180 (0.796–1.750)
Constant	−4.871	5.223	0.870	0.351	0.008

B = unstandardized regression coefficient; S.E., standard error; OR, odds ratio; CI, confidence interval; HEI, Healthy Eating Index; BMI, Body Mass Index; PM₁₀, Particulate Matter ≤10 μm; SO₂, Sulfur dioxide. **p < 0.001.

The overall model was significant (Omnibus χ^2^(8) = 80.609, *p* < 0.001; Nagelkerke *R*^2^ = 0.426). The Hosmer-Lemeshow test showed no statistically significant evidence of lack of fit (χ^2^(8) = 9.797, *p* = 0.280). The model demonstrated good discriminative ability (AUC = 0.853, 95% CI: 0.802–0.904), with sensitivity of 82.6%, specificity of 78.2%, PPV of 74.5%, and NPV of 85.3% at the optimal cut-off (Youden’s J = 0.61). Classification accuracy was 81.5%, compared to a baseline accuracy of 56.4% (predicting all as non-severe). Multicollinearity diagnostics (VIF range: 1.05–1.82; tolerance: 0.55–0.95) and the Box-Tidwell test (*p* > 0.05 for all continuous variables) indicated no violations of model assumptions.

### 3.6 ROC curve analysis for environmental parameters

Table 7 and Figure 1 present the results of the receiver operating characteristic (ROC) curve analysis evaluating the ability of environmental parameters to discriminate between severe (≥2+) and non-severe (<2+) ketonuria. Ambient temperature demonstrated the strongest discriminative ability, with an AUC of 0.799 (95% CI: 0.736–0.863, *p* < 0.001). A cut-off value of ≤15.75 °C yielded a sensitivity of 77.2% and a specificity of 79.0%, with a Youden’s J index of 0.562. PM₁₀ also showed good and statistically significant discriminative ability, with an AUC of 0.754 (95% CI: 0.689–0.818, *p* < 0.001). A cut-off value of >91.46 μg/m^3^ yielded a sensitivity of 62.0% and a specificity of 79.8%, corresponding to a Youden’s J index of 0.418. In contrast, SO₂ did not demonstrate statistically significant discriminative ability (AUC = 0.532, 95% CI: 0.452–0.612, *p* = 0.423); therefore, no optimal cut-off value was reported for this variable. Overall, ambient temperature showed the highest discriminatory performance among the environmental parameters examined, followed by PM₁₀. The cut-off values were optimized within the same dataset and should therefore be interpreted as exploratory rather than definitive clinical thresholds.

**Table 7:** Receiver operating characteristic (ROC) curve analysis of environmental parameters for discriminating severe (≥2+) versus non-severe (<2+) ketonuria.

Parameter	Cut-off value	AUC	Std. Error	*p*-value	95% CI	Sensitivity (%)	Specificity (%)	Youden index (J)
Ambient Temp. (°C)	≤15.75	0.799	0.032	<0.001**	0.736–0.863	77.2	79	0.562
PM10 (μg/m^3^)	>91.46	0.754	0.033	<0.001**	0.689–0.818	62	79.8	0.418
SO_2_ (μg/m^3^)	—	0.532	0.041	0.423	0.452–0.612	—	—	—

AUC, Area Under the Curve; CI, Confidence Interval; Std. Error, Standard Error; PM10, Particulate Matter <10 μm; SO_2_, Sulfur dioxide. This analysis compares severe (≥2+) versus non-severe (<2+) ketonuria. No ketonuria-negative participants were included because absence of ketonuria was an exclusion criterion. **p < 0.001.

**Figure 1:**
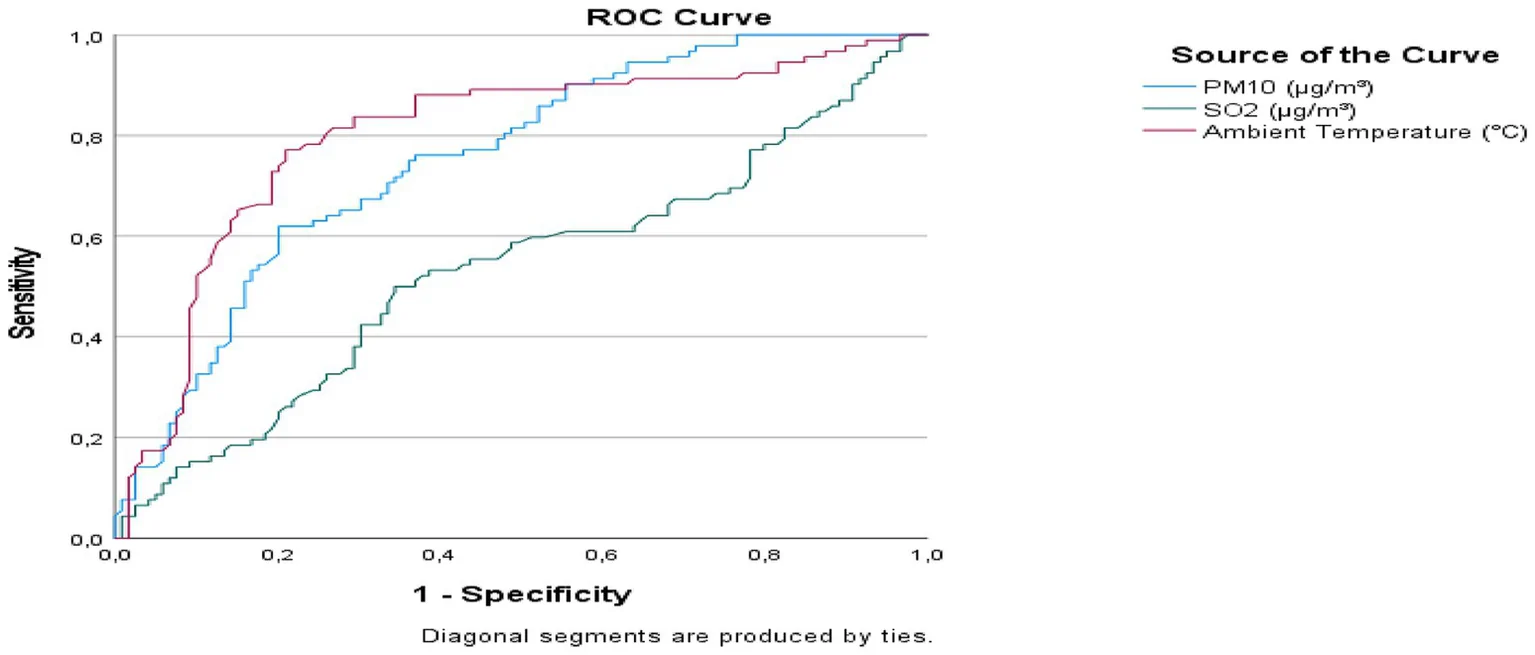
Receiver operating characteristic (roc) curves for environmental parameters in predicting severe ketonuria.

## 4 Discussion

### 4.1 Main findings

The present study demonstrates that lower ambient temperature and higher maternal age were independently associated with severe ketonuria in women with HG, after adjusting for multiple confounders. In contrast, PM₁₀ and SO₂, despite showing univariate associations, did not remain independent predictors. This suggests that ambient temperature may be a more relevant environmental factor associated with HG severity than individual air pollutants in this industrial region.

### 4.2 Seasonal dynamics and environmental exposure

The study setting, Batman, is an industrial city with a major oil refinery (TÜPRAŞ) and petroleum operations (TPAO), leading to high PM₁₀ levels that exceed EU limits (18). In winter, atmospheric inversions trap pollutants and reduce vertical mixing, leading to higher PM₁₀ concentrations (19, 20, 31). The present data confirm that PM₁₀ was significantly higher during autumn/winter than spring/summer. However, after adjusting for temperature and other covariates, PM₁₀ did not independently predict severe ketonuria (30). This finding aligns with recent evidence that the relationship between air pollution and HG may be non-causal or confounded by seasonality (12, 24). The association with ambient temperature may reflect underlying physiological effects of cold exposure (e.g., increased sympathetic activation or altered gastrointestinal motility) (26, 28); however, this remains a hypothesis, as thermal stress itself was not directly measured in this study and requires targeted physiological investigations for confirmation (32).

### 4.3 Comparison with previous literature

Studies examining air pollution and HG risk have reported inconsistent results. Zhou et al. (11) identified SO₂ as a risk factor, whereas the present study did not find SO₂ to be independently associated with severity. This discrepancy may be due to differences in outcome (risk vs. severity), exposure windows, or population characteristics similarly (36), the null findings for PM₁₀ in the present study are consistent with Mendelian randomization studies that failed to establish a causal link (12). Regarding temperature, previous research has linked extreme cold to adverse pregnancy outcomes (13, 14, 38), and the present findings extend this to HG severity. The mean winter temperature in the study cohort (11.45 °C) was close to the extreme cold thresholds identified in population studies (16), reinforcing the clinical relevance of cold exposure.

The present result salso highlight the importance of maternal age as a risk factor for severe HG. While older maternal age has been associated with HG risk (21), the present study is among the first to show its independent association with ketonuria severity. The high correlation between gravida and ketonuria in univariate analysis (*r_s_* = 0.450) attenuated after adjustment, suggesting that the effect of gravida may be mediated by age or seasonality.

### 4.4 Ketonuria as a severity Indicator

Ketonuria has been a controversial marker of HG severity. Koot et al. (22) found that ketonuria did not correlate with symptom severity or quality of life, while others have used it to stratify patients (23). In the present study, all participants had ketonuria by definition, so the outcome reflects (27) the degree of ketonuria (≥2 + vs. <2+) among patients with ketonuria. This binary classification was chosen for clinical practicality and has been used in previous studies (23, 34, 35). We acknowledge that ketonuria may not fully capture the multifaceted nature of HG severity; thus, the present findings should be interpreted with caution and future studies should incorporate validated symptom scores such as PUQE.

### 4.5 Strengths and limitations

#### 4.5.1 Strengths

We used daily environmental data matched to admission dates, reducing exposure misclassification relative to monthly averages. We adjusted for multiple covariates including nutritional status, and we performed thorough model diagnostics.

Limitations

- Retrospective design: We lacked standardised symptom severity scores and relied on ketonuria.
- Single-centre setting: Findings may not generalise to other regions.
- Exposure assessment: Station-level measurements do not capture individual micro-exposures (indoor, residential location, time outdoors). We also did not have data on socioeconomic status, smoking, or heating sources, which may confound the temperature–severity association.
- Exposure window: Same-day exposure may not reflect the relevant gestational period. Future studies should consider 1-, 3-, or 7-day moving averages.
- Seasonal confounding: Gestational week was strongly associated with season; although adjusted, residual confounding may persist.
- Statistical methods: We used traditional regression, not mixture models (e.g., Weighted Quantile Sum [WQS] or Bayesian Kernel Machine Regression [BKMR]) that can better account for correlated pollutants. Given the multicollinearity between temperature and PM₁₀, mixture approaches might have provided more nuanced insights. This is an area for future research.

### 4.6 Clinical and public health implications

While these findings are exploratory, they suggest that during colder months, clinicians should be particularly vigilant about HG severity, especially in older women. Public health interventions, such as warming shelters or targeted nutritional support during winter, may be beneficial; however, prospective studies are needed to evaluate their effectiveness. Women’s health nurses, given their central role in direct clinical care and continuous patient contact, are well positioned to monitor symptom severity, educate patients about environmental risk factors, and promote indoor warmth during cold spells. Close collaboration with obstetricians and primary care nurses and midwives, who often provide the first point of contact for pregnant women, could further strengthen early identification and management of environmental risk factors associated with HG severity.

## 5 Conclusion

This single-centre retrospective study found that lower ambient temperature and higher maternal age were independently associated with severe ketonuria in women with hyperemesis gravidarum. In contrast, PM₁₀, SO₂, and gravida were not independent predictors after adjustment. These findings, while requiring confirmation in larger prospective studies with individual-level exposure assessment, highlight ambient temperature as a potentially modifiable environmental factor in HG management. No causal conclusions should be drawn; the study demonstrates associations only.

## Data Availability

The raw data supporting the conclusions of this article will be made available by the authors, without undue reservation.

## Footnotes

1
https://sim.csb.gov.tr/SERVICES/airquality

## References

[1] Committee on Practice Bulletins-Obstetrics. ACOG practice bulletin no. 189: nausea and vomiting of pregnancy. Obstet Gynecol. (2018) 131:e15–30. doi: 10.1097/AOG.0000000000002456, 29266076

[2] Liu C Zhao G Qiao D Wang L He Y Zhao M . Emerging progress in nausea and vomiting of pregnancy and hyperemesis gravidarum: challenges and opportunities. Front Med. (2022) 8:809270. doi: 10.3389/fmed.2021.809270, 35083256 PMC8785858

[3] Vadakekut ES Mahdy H. Hyperemesis Gravidarum. In: StatPearls [Internet]. StatPearls Publishing; (2025). https://www.ncbi.nlm.nih.gov/books/NBK532917/ (Accessed January 15, 2026). 30422512

[4] Fejzo MS Trovik J Grooten IJ Sridharan K Roseboom TJ Vikanes Å . Nausea and vomiting of pregnancy and hyperemesis gravidarum. Nat Rev Dis Primers. (2019) 5:62. doi: 10.1038/s41572-019-0110-3, 31515515

[5] Fan J Yin M. Offspring of women with hyperemesis gravidarum are more likely to have cardiovascular abnormalities. BMC Pregnancy Childbirth. (2024) 24:119. doi: 10.1186/s12884-024-06293-6, 38331740 PMC10854153

[6] Hinkle SN Mumford SL Grantz KL Silver RM Mitchell EM Sjaarda LA . Association of nausea and vomiting during pregnancy with pregnancy loss: a secondary analysis of a randomized clinical trial. JAMA Intern Med. (2016) 176:1621–7. doi: 10.1001/jamainternmed.2016.5641, 27669539 PMC6191846

[7] Liu D Zhou K. The impact of hyperemesis gravidarum on fetal development and birth outcomes: a systematic review and meta-analysis. Clin Exp Obstet Gynecol. (2024) 51:197. doi: 10.31083/j.ceog5109197

[8] Cao M Kang Y Li J Gu J Liu L He J . Relationship between exposure to air pollutants in the first trimester and spontaneous abortion in pregnant women in the river valley city. Sci Rep. (2024) 14:27609. doi: 10.1038/s41598-024-76181-8, 39528500 PMC11555332

[9] Li Y Yang Z. The causal effect of exposure to air pollution on risk of adverse pregnancy outcomes: a two-sample Mendelian randomisation study. Sci Total Environ. (2024) 928:172234. doi: 10.1016/j.scitotenv.2024.172234, 38615756

[10] Nazarpour S Ramezani Tehrani F Valizadeh R Amiri M. The relationship between air pollutants and gestational diabetes: an updated systematic review and meta-analysis. J Endocrinol Investig. (2023) 46:1317–32. doi: 10.1007/s40618-023-02037-z 36807891

[11] Zhou Y Tao Q Liu Q Huang Q Song M Wang Q . The association between air pollutant exposures and hyperemesis Gravidarum. J Hazard Mater. (2026) 507:141726. doi: 10.1016/j.jhazmat.2026.141726, 41833165

[12] Shan C Chen L Mo H Chen X Han C Tao F . Preliminary study of air pollution and adverse pregnancy outcomes: a Mendelian randomization study. Atmosphere. (2024) 15:1285. doi: 10.3390/atmos15111285, 30654563

[13] Calle-Martínez A Ruiz-Páez R Gómez-González L Egea-Ferrer A López-Bueno JA Díaz J . Short-term effects of tropospheric ozone and other environmental factors on emergency admissions due to pregnancy complications: a time-series analysis in the Madrid region. Environ Res. (2023) 231:116206. doi: 10.1016/j.envres.2023.116206, 37217123

[14] Lin Y Hu W Xu J Luo Z Ye X Yan C . Association between temperature and maternal stress during pregnancy. Environ Res. (2017) 158:421–30. doi: 10.1016/j.envres.2017.07.005 28689033

[15] Akgun N Misirligil S Yegin GF Keskin HL Yavuz AF. Do meteorological variations and climate changes affect hyperemesis gravidarum? Z Geburtshilfe Neonatol. (2022) 226:304–10. doi: 10.1055/a-1808-1800, 35772724

[16] Dal Y Karagün Ş Çolak H Coşkun A. Seasonal variation of hyperemesis gravidarum prevalence. Perinatal J. (2023) 31:157–63. doi: 10.59215/prn.23.0312013, 35772724

[17] Tsigga M Filis V Hatzopoulou K Kotzamanidis C Grammatikopoulou MG. Healthy eating index during pregnancy according to pre-gravid and gravid weight status. Public Health Nutr. (2011) 14:290–6. doi: 10.1017/S1368980010001989, 20642871

[18] Ministry of Environment, Urbanization and Climate Change of Türkiye Türkiye Environmental Indicators 2025 General Directorate of Environmental Impact Assessment, Permits and Supervision (2025). Accessed September 12, 2025. Available online at: https://webdosya.csb.gov.tr/db/ced/icerikler/cevresel-gostergeler-2024_rev-ze-20251017093130.pdf (Accessed September 12, 2025)

[19] Bodor Z Bodor K Keresztesi Á Szép R. Major air pollutants seasonal variation analysis and long-range transport of PM10 in an urban environment with specific climate condition in Transylvania (Romania). Environ Sci Pollut Res. (2020) 27:38181–99. doi: 10.1007/s11356-020-09838-2, 32617823 PMC7496053

[20] Kida M Ziembowicz S. The impact of seasonality on air quality in terms of pollution with substances hazardous to the environment. Appl Sci. (2025) 15:6551. doi: 10.3390/app15126551

[21] Ashebir G Nigussie H Glagn M Beyene K Getie A. Determinants of hyperemesis gravidarum among pregnant women attending health care service in public hospitals of southern Ethiopia. PLoS One. (2022) 17:e0266054. doi: 10.1371/journal.pone.0266054, 35472152 PMC9042275

[22] Koot MH Grooten IJ Post JAMV Bais JMJ Ris-Stalpers C Naaktgeboren CA . Ketonuria is not associated with hyperemesis gravidarum disease severity. Eur J Obstet Gynecol Reprod Biol. (2020) 254:315–20. doi: 10.1016/j.ejogrb.2020.08.014, 33045502

[23] Aslan MM Yeler MT Bıyık İ Yuvacı HU Cevrioğlu AS Özden S. Hematological parameters to predict the severity of hyperemesis Gravidarum and Ketonuria. Rev Bras Ginecol Obstet. (2022) 44:458–66. doi: 10.1055/s-0042-1743101, 35405756 PMC9948287

[24] Aguilera J Konvinse K Lee A Maecker H Prunicki M Mahalingaiah S . Air pollution and pregnancy. Semin Perinatol. (2023) 47:151838. doi: 10.1016/j.semperi.2023.151838, 37858459 PMC10843016

[25] Clark SM Zhang X Goncharov DA. Inpatient management of hyperemesis gravidarum. Obstet Gynecol. (2024) 143:745–58. doi: 10.1097/AOG.0000000000005518, 38301258

[26] Dean CR Bruin CM O'Hara ME Roseboom TJ Leeflang MM Spijker R . The chance of recurrence of hyperemesis gravidarum: a systematic review. Eur J Obstet Gynecol Reprod Biol X. (2019) 5:100105. doi: 10.1016/j.eurox.2019.100105, 32021976 PMC6994404

[27] Dehdashti B Bagheri N Amin MM Hajizadeh Y. Impacts of climate changes on pregnancy and birth outcomes: a review. Int J Environ Health Eng. (2020) 9:24. doi: 10.4103/ijehe.ijehe_37_19

[28] Fassett MJ Peltier MR Lopez AH Chiu VY Getahun D. Hyperemesis gravidarum: risk of recurrence in subsequent pregnancies. Reprod Sci. (2023) 30:1198–206. doi: 10.1007/s43032-022-01078-8, 36163577

[29] Geodatos. (n.d.) Geographic coordinates of Batman, Türkiye. Available online at: https://www.geodatos.net/en/coordinates/turkey/batman (Accessed October 10, 2025)

[30] Li J Gu J Liu L Cao M Wang Z Tian X . The relationship between air pollutants and preterm birth and blood routine changes in typical river valley city. BMC Public Health. (2024) 24:1677. doi: 10.1186/s12889-024-19140-2, 38915004 PMC11197378

[31] Madronich S Sulzberger B Longstreth JD Schikowski T Andersen MPS Solomon KR . Changes in tropospheric air quality related to the protection of stratospheric ozone in a changing climate. Photochem Photobiol Sci. (2023) 22:1129–76. doi: 10.1007/s43630-023-00369-6, 37310641 PMC10262938

[32] McMichael AJ Woodruff RE Hales S. Climate change and human health: present and future risks. Lancet. (2006) 367:859–69. doi: 10.1016/S0140-6736(06)68079-3 16530580

[33] Ministry of Environment, Urbanization and Climate Change of Türkiye Türkiye Environmental Problems and Priorities Assessment Report Ministry of Environment, Urbanization and Climate Change (2023) Available online at: https://webdosya.csb.gov.tr/db/ced/icerikler/turk-ye-cevre-sorunlari-ve-oncel-kler-_2022_3_ver3.logoduzenlendi-20230901135641.pdf (Accessed September 12, 2025)

[34] Nana M Painter R Williamson C Nelson-Piercy C. Hyperemesis gravidarum. Lancet. (2026) 407:78–89. doi: 10.1016/S0140-6736(25)01454-0 41242332

[35] Nelson-Piercy C Dean C Shehmar M Gadsby R O'Hara M Hodson K . The management of nausea and vomiting in pregnancy and hyperemesis gravidarum (green-top guideline no. 69). BJOG. (2024) 131:e1–e30. doi: 10.1111/1471-0528.17739, 38311315

[36] Parasin N Amnuaylojaroen T Saokaew S. Prenatal PM2.5 exposure and its association with low birth weight: a systematic review and meta-analysis. Toxics. (2024) 12:446. doi: 10.3390/toxics12070446, 39058098 PMC11280910

[37] Vinikoor-Imler LC Davis JA Meyer RE Messer LC Luben TJ. Associations between prenatal exposure to air pollution, small for gestational age, and term low birthweight in a state-wide birth cohort. Environ Res. (2014) 132:132–9. doi: 10.1016/j.envres.2014.03.040, 24769562

[38] Qiao S Guo Q He Z Feng G Wang Z Li X. Spatiotemporal trends and drivers of PM2.5 concentrations in Shandong Province from 2014 to 2023 under socioeconomic transition. Toxics. (2025) 13:978. doi: 10.3390/toxics13110978, 41304529 PMC12656645

